# A bioavailable form of curcumin suppresses cationic host defence peptides cathelicidin and calprotectin in a murine model of collagen-induced arthritis

**DOI:** 10.1186/s13075-023-03148-x

**Published:** 2023-09-04

**Authors:** Mahadevappa Hemshekhar, Dylan Lloyd, Hani El-Gabalawy, Neeloffer Mookherjee

**Affiliations:** 1 Manitoba Centre for Proteomics and Systems Biology, Department of Internal Medicine, University of Manitoba, 799 John Buhler Research Centre, 715 McDermot Ave, Winnipeg, MB R3E3P4 Canada; 2 Division of Rheumatology, Department of Internal Medicine, University of Manitoba, Winnipeg, MB Canada; 3 Department of Immunology, University of Manitoba, Winnipeg, MB Canada

**Keywords:** Arthritis, Inflammation, Host defence peptides, Curcumin, Cathelicidin, Calprotectin

## Abstract

Curcumin, a component of the South-Asian spice turmeric, elicits anti-inflammatory functions. We have previously demonstrated that a highly bioavailable formulation of cucurmin, Cureit/Acumin™ (CUR), can suppress disease onset and severity, in a collagen-induced arthritis (CIA) mouse model. In a previous study, we have also shown that the abundance of antimicrobial host defence peptides, specifically cathelicidin (CRAMP) and calprotectin (S100A8 and S100A9), is significantly increased in the joint tissues of CIA mice. Elevated levels of cathelicidin and calprotectin have been associated with the pathogenesis of rheumatoid arthritis. Therefore, in this study, we examined the effect CUR administration on the abundance of cathelicidin and calprotectin in the joints, in a CIA mouse model. Here, we demonstrate that daily oral administration of CUR significantly reduces the elevated levels of CRAMP and calprotectin to baseline in the joints of CIA mice. We also show a linear correlation between the abundance of these peptides in the joints with serum inflammatory cytokines TNFα, IFNγ, and MCP-1. Overall, our results suggest that oral administration of a bioavailable CUR can suppress cathelicidin and calprotectin in the joints and regulate both local (joints) and systemic (serum) inflammation, in inflammatory arthritis.

## Brief report of research findings

Curcumin (CUR) is a component of turmeric, a South-Asian spice, shown to exhibit anti-inflammatory effects [1]. However, a major challenge in its use is its limited bioavailability [2]. In a previous study, we demonstrated the beneficial effects of a highly bioavailable form of curcuminoid formulation (Cureit/Acumin™) in a collagen-induced arthritis (CIA) mouse model. We showed that Cureit™ curcumin (CUR) delays disease onset and decreases clinical symptoms and the abundance of circulating inflammatory cytokines, in the CIA mice [3]. We have also established a panel of proteins that are significantly elevated in the joint tissues of CIA mice, which includes cationic host defence peptides (CHDP), specifically cathelicidin CRAMP and calprotectin (S100A8 and S100A9) [4]. CHPD, also known as antimicrobial peptides, are endogenous peptides that modulate hosts’ immune responses to control infection and inflammation [5, 6]. These peptides elicit both pro- and anti-inflammatory effects; however, their role in chronic inflammatory and autoimmune diseases such as arthritis remains ambiguous [5, 7, 8]. Elevated levels of the human cathelicidin peptide LL-37, and calprotectin, have been demonstrated in the serum and synovial joints of rheumatoid arthritis (RA) patients [8–10]. Elevated levels of cathelicidin and calprotectin are associated with the pathogenic effects of arthritis in both humans and mice [9–12]. Therefore, in this study, we examined the effects of CUR administration on the abundance of cathelicidin CRAMP and calprotectin (S100A8 and S100A9) in the joints of CIA mice. Here, we show that oral administration of CUR significantly reduces the abundance of CRAMP and calprotectin in the joints of CIA mice. We also demonstrate a linear correlation between the abundance of these peptides in the joints of CIA mice with circulating inflammatory cytokines. Overall, the results of this study demonstrates that the highly bioavailable CUR suppresses cathelicidin and calprotectin peptides in the joints of CIA mice to baseline levels and indicate that CUR can regulate both local and systemic inflammation in CIA mice.

## Methods

### Collagen-induced arthritis (CIA) mouse model

In this study, we have used a synchronized CIA murine model previously described by us [3, 13], approved by The University of Manitoba Animal Research Ethics Board. Experimental design and reporting of data are compliant with the ARRIVE guidelines for in vivo animal research. Briefly, male DBA/1 J mice (∼ 6 weeks old) obtained from Jackson laboratories were fed with a standard diet [3, 14]. Either bioavailable CUR (Cureit/Acumin™; Aurea Biolabs Ltd, Kerala, India) at a dose of 100 mg/kg per mouse or saline (100 μl) was administered daily by oral gavage. Mice were challenged with bovine collagen type II (CII) after 2 weeks of acclimatization as previously described by us [3, 13]. CIA challenge and CUR administration were performed between 10 am and 1 pm on specified days. Based on our previous studies, joint tissues and blood were collected on day 29 after the first CII challenge, after the appearance of clinical symptoms and elevated inflammatory markers in joints and serum [3]. One hind-paw joint from each mouse was homogenized to obtain protein lysates.

### Immunoblotting

Joint tissue lysates (25 μg) were resolved on NuPage 4–12% Bis–Tris protein gels (Invitrogen) and transferred onto nitrocellulose membranes. The membranes were blocked overnight with 5% milk powder (w/v) in TBST (20 mM Tris–HCl pH 7.5, 150 mM NaCl, 0.1% Tween-20) and probed with antibodies for murine CRAMP, S100A8, and S100A9 (Abcam, USA). Antibody to β-actin (Cell Signaling Technologies) was used to normalize for protein loading. Affinity-purified horseradish peroxidase (HRP)-linked secondary antibodies (Cell Signaling, USA) along with Amersham ECL Prime (GE Healthcare) was used for detection. The blots were imaged using AmershamTM Imager 680 blot and gel imager. Densitometry assessment of band intensity was determined using the AmershamTM Imager 680 analysis software version 2.0. The relative band intensity was assessed after normalization with the band intensity for β-actin.

### Evaluation of cytokines

Serum cytokine and chemokines were measured using the V-Plex mouse cytokine 29-Plex kit by Meso Scale Discovery (MSD) Platform (Meso Scale Diagnostics, Rockville, MD, USA), according to the manufacturer’s instructions. In this study, the levels of TNFα, IFNγ, and MCP-1 in serum were used to perform correlation analysis with the abundance of the peptides CRAMP and calprotectin (S100A8/A9) in the joints.

### Statistical analysis

The GraphPad Prism version 9 software was used for data analyses. The statistical significance was determined by Kruskal–Wallis one-way analysis of variance (ANOVA) followed by Dunn’s multiple comparison post hoc test. Pearson’s correlation analysis was performed to examine the correlation between the abundance of inflammatory cytokines and host defense peptides. A *p*-value of ≤ 0.05 was considered to be statistically significant.

## Results

### CUR significantly suppresses the abundance of CRAMP and calprotectin in the joints of CIA mice

**Fig. 1:**
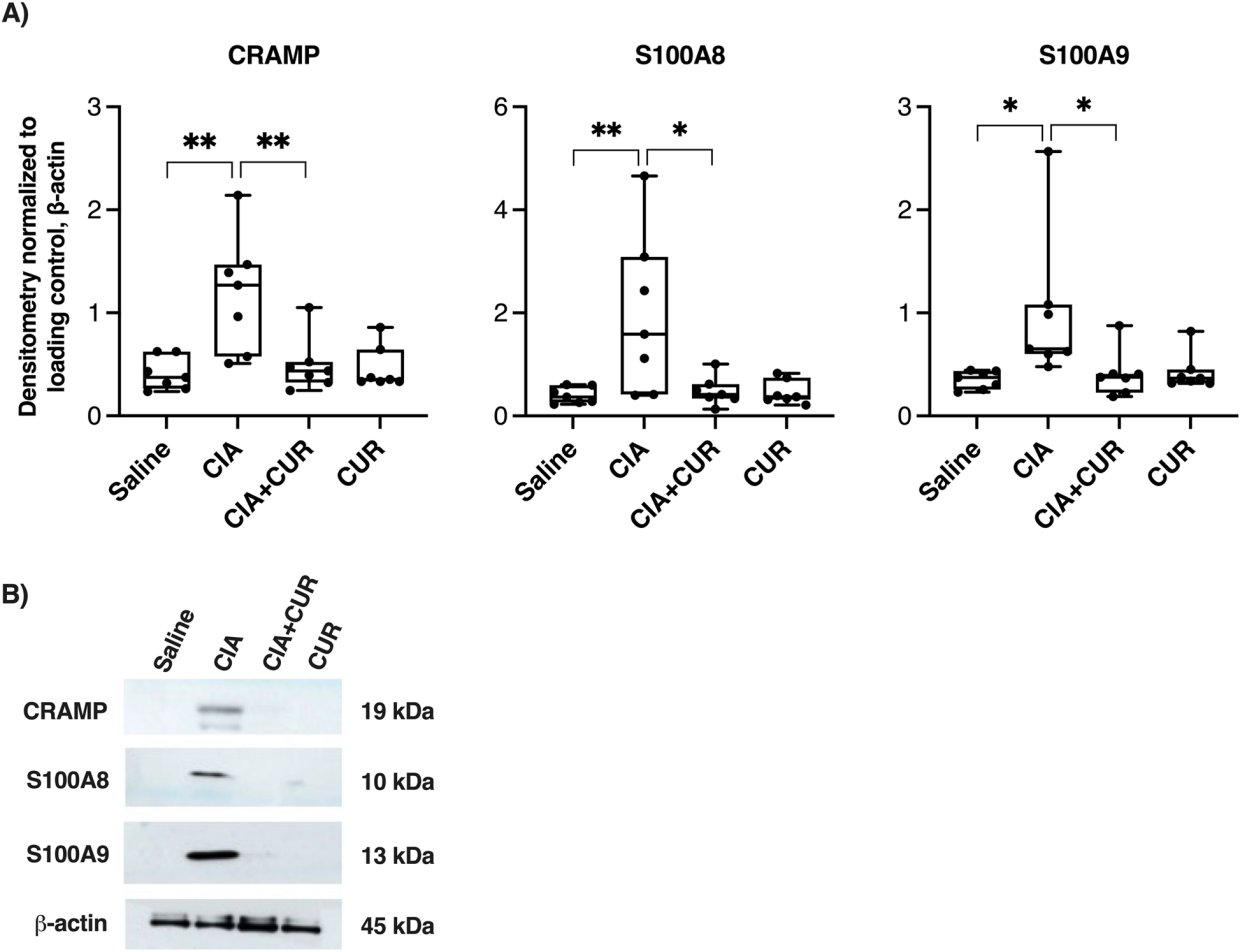
CUR reduces CRAMP and Calprotectin levels in the joints of CIA mice. Joint tissues obtained from four different groups of mice, CIA mice, saline control mice, CIA mice with oral administration of CUR, and CUR alone control mice (*n* = 7 per group) were homogenized. Joint tissue lysates (25 μg protein each) were resolved on NuPage 4–12% Bis–Tris protein gels and probed in immunoblots to assess the abundance of mouse CRAMP, S100A8, and S100A9. Antibody for β-actin was used to assess loading control. **A.** Densitometry for band intensity was determined using the AmershamTM Imager 680 analysis software version 2.0. The relative band intensity was determined by normalizing to the β-actin band intensity for each sample. The GraphPad Prism 9 software was used for statistical analyses. One-way ANOVA with Tuckey’s post hoc multiple comparison analysis was used to determine the *p*-values (*p ≤ 0.05, **p ≤ 0.005). **B.** A western blot image representative of data obtained from *n* = 7 mice per group

Consistent with our previous study [4], joint tissue lysates of CIA mice showed significantly elevated levels of CRAMP and calprotectin (S100A8 and S100A9) compared to saline control mice (Fig. 1). Oral administration of CUR significantly reduced the levels of CRAMP (by 85 ± 28%), S100A8 (by 90 ± 14%), and S100A9 (by 84 ± 29%) in the joint tissue lysates of CIA mice, restoring the levels back to baseline (Fig. 1).

### Cathelicidin and calprotectin peptides in the joints correlate with circulating inflammatory cytokines

**Fig. 2:**
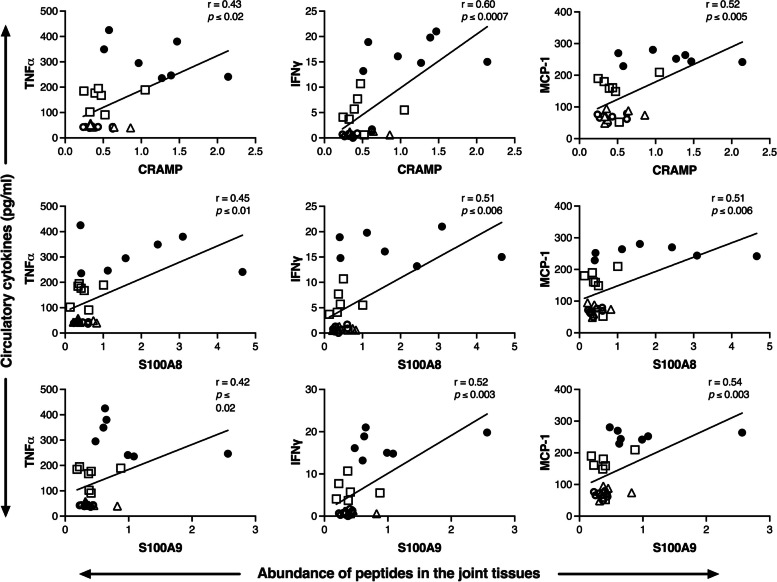
Correlation between abundance of CRAMP, S100A8, and S100A9 in the joints with circulating serum levels of TNFα, IFNγ, and MCP-1 in the serum. The abundance of CRAMP, S100A8, and S100A9 in the joints were determined by western blot densitometry. The serum levels of TNFα, IFNγ, and MCP-1 was determined by Meso Scale Discovery platform. The GraphPad Prism 9 software was used for statistical analyses. Pearson’s correlation analysis was performed to determine the correlation between the abundance of each of the peptides in the joints with the levels of TNFα, IFNγ, and MCP-1 in the serum. Each data point represents a single mouse, from *n* = 7 per group; saline control (○), CIA mice (●), CIA mice with CUR (□), and CUR alone (Δ)

We had previously demonstrated that the abundance of inflammatory cytokines TNFα, IFNγ, and MCP-1 is significantly elevated in the serum of CIA mice and that the oral administration of CUR significantly reduces the serum levels of these cytokines [3]. Here, we demonstrate that the abundance of CRAMP, S100A8, and S100A9 in the joint tissues exhibits a linear correlation with serum inflammatory cytokines TNFα, IFNγ, and MCP-1 (Fig. 2). Taken together, these results indicate that CUR-mediated decrease of CRAMP, S100A8, and S100A9 in the joints (Fig. 2) correlates with CUR-mediated decrease of circulating inflammatory cytokines [3].

## Discussion

In this study, we demonstrate that the oral administration of a highly bioavailable form of curcumin, CUR (Cureit/Acumin™), significantly reduces the elevated levels of mouse CHDPs cathelicidin (CRAMP) and calprotectin (S100A8 and S100A9) in the joint tissues of CIA mice. To our knowledge, this is the first study to demonstrate the ability of CUR to modulate the levels of CHDP levels in joint tissues in inflammatory arthritis. We have previously shown that oral administration of CUR significantly decreases the abundance of pro-inflammatory cytokines TNFα, IFNγ, and MCP-1 in CIA mice [3]. Here, we demonstrate a linear correlation between CHDPs, CRAMP, and calprotectin and abundance in the joints with the serum levels of TNFα, IFNγ, and MCP-1. Taken together, these results indicate that the oral administration of CUR modulates both local (joints) and systemic (serum) molecular markers associated with inflammatory arthritis. Overall, the findings reported in this study adds to the limited understanding of the immunomodulatory effects of a bioavailable form of curcumin in inflammatory arthritis.

Cathelicidin and calprotectin peptides influences innate and adaptive immunity, and facilitate both pro- and anti-inflammatory responses depending on the cellular milieu and disease state [5, 8, 15]. Cathelicidins can mediate classical pro-inflammatory responses such as chemokine secretion, chemotaxis, and polarization and maturation of dendritic and T cells [5]. In contrast, cathelicidins can also suppress pro-inflammatory cytokine production and cytokine-mediated and/or endotoxin-induced inflammatory responses [5, 7, 8]. Similarly, calprotectin can mediate pro-inflammatory functions by initiating chemotaxis and in contrast can also act as an anti-inflammatory agent primarily by exhibiting oxidant scavenging activity [15]. Human studies indicate that the levels of these peptides are associated with disease severity in inflammatory arthritis. For example, levels of the human cathelicidin LL-37 and calprotectin (S100A8/A9) are enhanced in the serum and synovium of RA patients [10, 16, 17]. Similarly, previous studies have demonstrated elevated levels of cathelicidin (mouse CRAMP) and calprotectin peptides in murine models of arthritis [4, 18]. Furthermore, reduction of joint inflammation shows a correlative decrease in the serum levels of LL-37 and calprotectin in RA patients following anti-inflammatory treatments such as methotrexate, glucocorticoid, and sulphasalazine [17]. Taken together, these studies indicate that cathelicidin and calprotectin may be indicators of disease severity in inflammatory arthritis. This is further corroborated by our results, wherein CRAMP and calprotectin are significantly elevated in the joint tissues of CIA mice and significantly suppressed by the oral administration of CUR which is an anti-inflammatory agent [3, 19–21]. Our results demonstrate that the oral administration of CUR restores the elevated levels of cathelicidin and calprotectin to baseline in the joints of CIA mice. In addition, we show a linear correlation between the abundance of cathelicidin and calprotectin in the joints with circulating inflammatory cytokines in serum.

Inflammatory cytokines such as TNFα, IFNγ, and MCP-1 play a key role in the development and pathogenesis of RA by engaging critical immune signaling cascades NF-κB, STAT, and AP1, in antigen-presenting cells such as macrophages and dendritic cells [22]. CUR modulates innate immune responses by mitigating the production of critical cytokines and chemokines including TNFα, IFNγ, and MCP-1 [22]. We have previously shown that the abundance of cathelicidin and calprotectin correlate with cellular influx in the joints of CIA mice [4]. Therefore, it is likely that oral administration of CUR may modulate responses mediated by leukocytes in the joints of CIA mice. It has been previously shown that CUR can inhibit the activation, proliferation, and differentiation of naïve CD4 + T cells to T helper (Th)1 and Th17 subtypes and prevent joint and bone destruction [23]. A previous study showed an association of elevated level of cathelicidin in the joints with serum inflammatory markers in a rat model of arthritis [10]. To our knowledge, this is the first study to demonstrate a linear correlation between the abundance of both cathelicidin and calprotectin in the joints with circulating serum levels of inflammatory cytokines in a CIA mouse model. Our results indicate that the elevated levels of these peptides in the joints may be associated with the systemic disease manifestation in inflammatory arthritis, both of which are significantly suppressed by the oral administration of CUR. Overall, CUR is a safe and cost-effective intervention that can effectively modulate both local and systemic immune responses in inflammatory arthritis.

## Data Availability

The datasets used and/or analyzed during the current study are available from the corresponding author on reasonable request. All data generated or analyzed during this study are included in this published article.

## Abbreviations

RA: Rheumatoid arthritis
CIA: Collagen-induced arthritis
CUR: Curcumin
CHDP: Cationic host defence peptides
CRAMP: Cathelicidin-related antimicrobial peptide
TBST: Tris-buffered saline with 0.1% Tween® 20 Detergent
HRP: Horseradish peroxidase
TNFα: Tumor necrosis factor alpha
IFNγ: Interferon gamma
MCP-1: Macrophage colony stimulating factor 1
Th: T helper
